# Immunotherapy for Refractory Autoimmune Encephalitis

**DOI:** 10.3389/fimmu.2021.790962

**Published:** 2021-12-16

**Authors:** Jiawei Yang, Xueyan Liu

**Affiliations:** Department of Pediatrics, Shengjing Hospital of China Medical University, Shenyang, China

**Keywords:** autoimmune encephalitis, refractory, third-line therapy, rituximab, neuroimmunology

## Abstract

Autoimmune encephalitis (AE) is an immune-mediated disease involving the central nervous system, usually caused by antigen-antibody reactions. With the advent of autoantibody-associated diseases, AE has become a hot research frontier in neuroimmunology. The first-line conventional treatments of autoimmune encephalitis consist of steroids, intravenous immunoglobulin (IVIG), plasma exchange (PLEX), and second-line therapy includes rituximab. Despite considerable research and expanding clinical experience, current treatments are still ineffective for a significant number of patients. Although there is no clear consensus, clinical trial evidence limited, and the level of evidence for some of the drugs based on single reports, third-line therapy is a viable alternative for refractory encephalitis patients. With the current rapid research progress, a breakthrough in the treatment of AE is critical. This article aims to review the third-line therapy for refractory AE

## Introduction

Autoimmune encephalitis (AE) was first reported in the 1960s (1). It was initially described as limbic encephalitis (LE), characterized by seizures, movement disorders, behavioral changes, mood disorders, cognitive impairment, autonomic dysfunction, and altered level of consciousness. The majority of LE was initially diagnosed with paraneoplastic syndromes commonly associated with lung, breast, ovarian, or testicular malignancies. The autoantibodies target nuclear and cytoplasmic proteins. The vast majority of these patients have a seemingly poor prognosis and do not respond to immunotherapy (2). A previous study has found that tumor treatment often improves neurological function, suggesting that autoantibodies play indirect pathogenic roles and changes in autoantibody levels may not achieve a complete cure (3). Therefore, LE is known as “paraneoplastic LE”. In recent years, autoantibodies targeting cell surfaces or ion channels, such as Anti-N-methyl-d-aspartate receptor (NMDAR) antibodies, have been identified. These autoantibodies affect not only the limbic system but also various brain structures (4). Most of these patients do not have malignancies and respond well to immunotherapy. The term AE gradually replaces autoimmune LE and specifically refers to the disease entity. In this context, AE refers to autoantibodies targeting cell surfaces or ion channels.

To date, the treatment of AE is mostly based on data from retrospective cohort and clinical cases, and prospective randomized controlled studies on immunotherapy are rare (5). The dosage and treatment course of AE are generally modeled on the treatment of rheumatism or other immune-mediated central nervous system diseases and refer to the clinical experience of experts. Unlike paraneoplastic LE, antibodies targeting cell surface receptors can be pathogenic, bind to receptors, and directly affect neuronal function rather than causing neuronal death. Animal models replicating features of AE by passive transfer of cerebrospinal fluid (CSF) or immunoglobulins from anti-NMDAR encephalitis patients to mice provided more direct evidence of autoantibody pathogenicity (6). Therefore, the pathogenic key is the continuous production of autoantibodies by self-reactive B cells and their subsequent proliferation and differentiation into autoantigen reactive memory B cells and autoantibody-secreting plasma cells. Autoantibodies against neuronal surfaces mediate pathogenic effects *via* multiple mechanisms. Observing the incubation of neuron culture with anti-NMDAR encephalitis patients’ autoantibody, patients’ antibodies cross-linking and internalizing NMDA receptors result in a selective and reversible decrease in NMDAR surface density and synaptic localization (7, 8). This is one of the main pathogenic pathways of autoantibodies. Another pathogenic pathway is complement activation, demonstrated in a case study of contactin-associated protein-like 2 (CASPR2) antibody-associated encephalitis (9).

Based on in-depth tumor screening, the immunotherapy framework for AE can be divided into first-line therapy and second-line therapy. First-line therapies include steroids, IVIG, and PLEX. They have a rapid onset of action with lower risk and could suppress autoimmune diseases systemically and non-specific. Thus, they are widely used as the first choice of treatment against AE. Steroids and IVIG are usually combined, which exhibit good efficacy in more than half of patients and are well tolerated, with few adverse effects that make treatment impossible to continue (10). PLEX is another option for patients with incomplete response to steroids or IVIG; however, it is limited due to being perceived as more invasive. PLEX would be more difficult to accept in children and in patients with poor co-operation (11). Previous studies have shown that timely delivery of immunotherapies is crucial (12). Delayed treatment due to negative autoantibodies can lead to poor prognosis and even death (13). Therefore, immunotherapy should be started once the clinical symptoms highly suspect AE and exclude infectious encephalitis (14). If first-line therapies are ineffective, physicians will choose second-line therapies, yet there is still controversy about the observation time limit. Second-line therapy includes the monoclonal antibody against CD20, i.e., rituximab, and is also preferentially considered by most clinical specialists (14). In a cohort study that included 27 cases of anti-NMDAR encephalitis and 3 cases of anti-leucine-rich glioma inactivated-1 (LGI1) encephalitis, mRS scores improved more in the rituximab treated group, both in the group that responded to first-line therapy (88% vs 83.3%) and in the group that did not respond to first-line therapy (60% vs 22.2%) (15). Moreover, adverse reactions caused by rituximab are mostly mild, and life-threatening severe adverse reactions are rare (16). The remainder includes cyclophosphamide, an alkylating agent that inhibits cell proliferation, as well as the oral antimetabolite drugs mycophenolate mofetil and azathioprine (17–19). Cyclophosphamide is often used as a monotherapy for rheumatic diseases affecting the CNS, such as mental lupus and primary CNS vasculitis (20). However, given its slow mechanism of action and significant side effects, it is less frequently used than rituximab to treat AE (21). Particularly, it is rarely used alone and mostly used in combination with rituximab. However, 19%-33% of patients with AE still do not respond well to first-line and second-line therapy, and there are persistent neurological and psychological problems (10, 22–24). New immunotherapy drugs, known as “third-line therapy”, are increasingly emerging for patients with refractory AE. This article is a narrative review of immunotherapy drugs for treating refractory AE (**Table 1**).

**Table 1:** Overview of third-line therapy drugs.

Name	Mechanism	Function	Side effects	Contraindications	Regimen
**Bortezomib**	Inducing cell-cycle arrest and apoptosis on long-live plasma cells by accumulating the misfolded proteins.	Depleting antibody-producing plasma cells, lowering the titers of autoantibodies	Infusion reactions, cytopenia, heart failure exacerbation, infection, herpes reactivation, acute lung injury and neuropathy	Allergy, heart failure and hypotension thrombocytopenia should be used with caution	Subcutaneous injection of 1.3 mg/m2, twice weekly for 2 weeks (days 1, 4, 8, and 11), followed by a 10-day rest
**Tocilizumab**	Blocking IL-6-mediated signal transduction	Inducing the differentiation and proliferation of B cells, keeping plasma cells alive, inducing helper T cell differentiation, and producing other cytokines, such as IL-17; stimulating cytotoxic T cells。	Infusion reactions, infection, neutropenia, hypertension	Allergy; severe infection; gastrointestinal perforation tuberculosis should be treated first if there is active or latent tuberculosis infection	8mg/kg, intravenous injection
			Meningoencephalitis; Cognitive impairment and leukoencephalopathy; autoimmune encephalitis		
**Daratumumab**	Inducing CD38-expressing plasma cell apoptosis	Depleting antibody-producing plasma cells, lowering the titers of autoantibodies	Fatigue, nausea, anemia; neutropenia, diarrhea and cough; Serious infection	Allergy; severe infection or severe impairment of immune responses	16 mg/kg, intravenous injection
**Tofacitinib**	A selective inhibitor of the JAK family of tyrosine kinases	Passing through the blood-brain barrier (BBB), modulating the immune response to a wide range of cytokine receptors	Neutropenia, headaches, diarrhea, fatigue, hypertension and symptoms of upper respiratory tract infection; severe infections, reactivation of latent tuberculosis, gastrointestinal perforation and *de novo* malignancies	Allergy; severe infection; gastrointestinal perforation tuberculosis should be treated first if there is active or latent tuberculosis infection	5 mg twice daily, oral
**Low-dose Interleukin 2**	Specifically activating regulatory T cells without stimulating effector T cells	Inhibiting the activation and proliferation of multiple immune cells and suppressing cytokine production	Injection site reactions, influenza-like symptoms, nausea, neutropenia, subclinical hyperthyroidism	Allergy; seizures, severe hypotension, severe cardiac and renal dysfunction, severe infections	Subcutaneous injection of 1.5 million IU/day for 5 days, followed by three 5-day courses of 3 million IU/day at weeks 3, 6, and 9
**Rapamycin**	Inhibiting T-cell-mediated immune response	Exhibiting both protective and therapeutic effects in injured central nervous system by reducing overzealous inflammatory responses	Headache, nausea, dizziness, epistaxis, joint pain, thrombocytopenia, leucopenia, hypertriglyceridemia, hypercholesterolemia, hyperglycemia, elevated liver enzymes	Allergy, severe infections	loading dose: 6mg/d oral
					maintenance dose: 3mg/d oral
					maintain trough concentrations of 8–12 ng/mL

Because refractory AE is uncommon, much of the data included in this review is low-level evidence, such as case reports and observational cohort studies. For further investigation, a higher level of evidence is required. The evidence of relevant articles has been summarized in **Table 2**.

**Table 2:** Studies on third-line therapy for refractory autoimmune encephalitis.

Name	Study Population	Result	Type	References
**Bortezomib**	A 22-year-old woman with refractory anti-NMDAR encephalitis	Clinical symptoms improved; autoantibody titers decreased from 1:1000 to 1:320 in serum	Case report	Schroeder Christoph et al. in (25).
	An 8-year-old girl with refractory anti-NMDAR encephalitis	Clinical symptoms improved; autoantibody titers decreased from 1:20 to 1:2 in CSF	Case report	Cordani R et al. in (26)
	5 cases of severe refractory anti-NMDA encephalitis	Three patients improved to a minimally conscious state; Two patients’ autoantibody titers decreased in CSF	Case series	Yong-Won Shin et al. in (27).
	A 26-year-old woman with refractory anti-NMDAR encephalitis	Clinical symptoms improved	Case report	Olafur Sveinsson et al. in (28).
	Two cases of severe refractory anti-NMDA encephalitis	Significant clinical improvement	Case series	S. Keddie et al. in (29).
**Tocilizumab**	A 64-year-old man with refractory Anti-CASPR2 Antibodies	Clinical and neuroradiological improved; autoantibody titers decreased from 1:1000 to 1:100 in serum	Case report	Maurizio benucci et al. in (30).
	Three children with refractory autoimmune encephalitis	Clinical symptoms improved	Case series	Randell R. et al. in (31)
	An 8-year-old girl with refractory anti-GAD encephalitis	Clinical symptoms improved; autoantibody levels declined from 303.1 U/ml to 30 U/ml in serum	Case report	Jaafar F. et al. in (32).
	30 patients with refractory autoimmune encephalitis	Higher frequencies of mRS improvement and favorable clinical responses compared with the control groups	Cohort study	Lee et al. in (33).
	52 patients with anti-NMDA encephalitis	Better efficacy than the control groups	Cohort study	Lee et al. in (34).
**Daratumumab**	A 60-year-old man with refractory anti-CASPR2 encephalitis	Clinical symptoms improved; autoantibody titers decreased from > 1:1.000 to 1:32 in CSF and > 1:10.000 to 1:10.000 in serum	Case report	Scheibe F et al. in (35).
	A 26-year-old woman with refractory anti-NMDAR encephalitis	Clinical symptoms improved	Case report	Ratuszny D et al. in (36).
**Tofacitinib**	8 patients with refractory autoimmune encephalitis	Two had good responses; three had partial responses; three showed no significant improvements	Cohort study	Lee et al. in (34).
**Low-dose Interleukin 2**	10 patients with refractory autoimmune encephalitis	Functional outcome improved in six patients	Cohort study	Lim JA et al. in (37).
**Rapamycin**	17 patients with anti-Hu encephalitis	Over half of the patients improved or stabilized functional disabilities	Cohort study	de Jongste AH et al. in (38).
	An 8-year-old girl with refractory anti-NMDAR encephalitis	Clinical symptoms improved	Case report	Cordani R et al. in (26)

## Third-Line Therapy for Refractory AE

**Figure 1:**
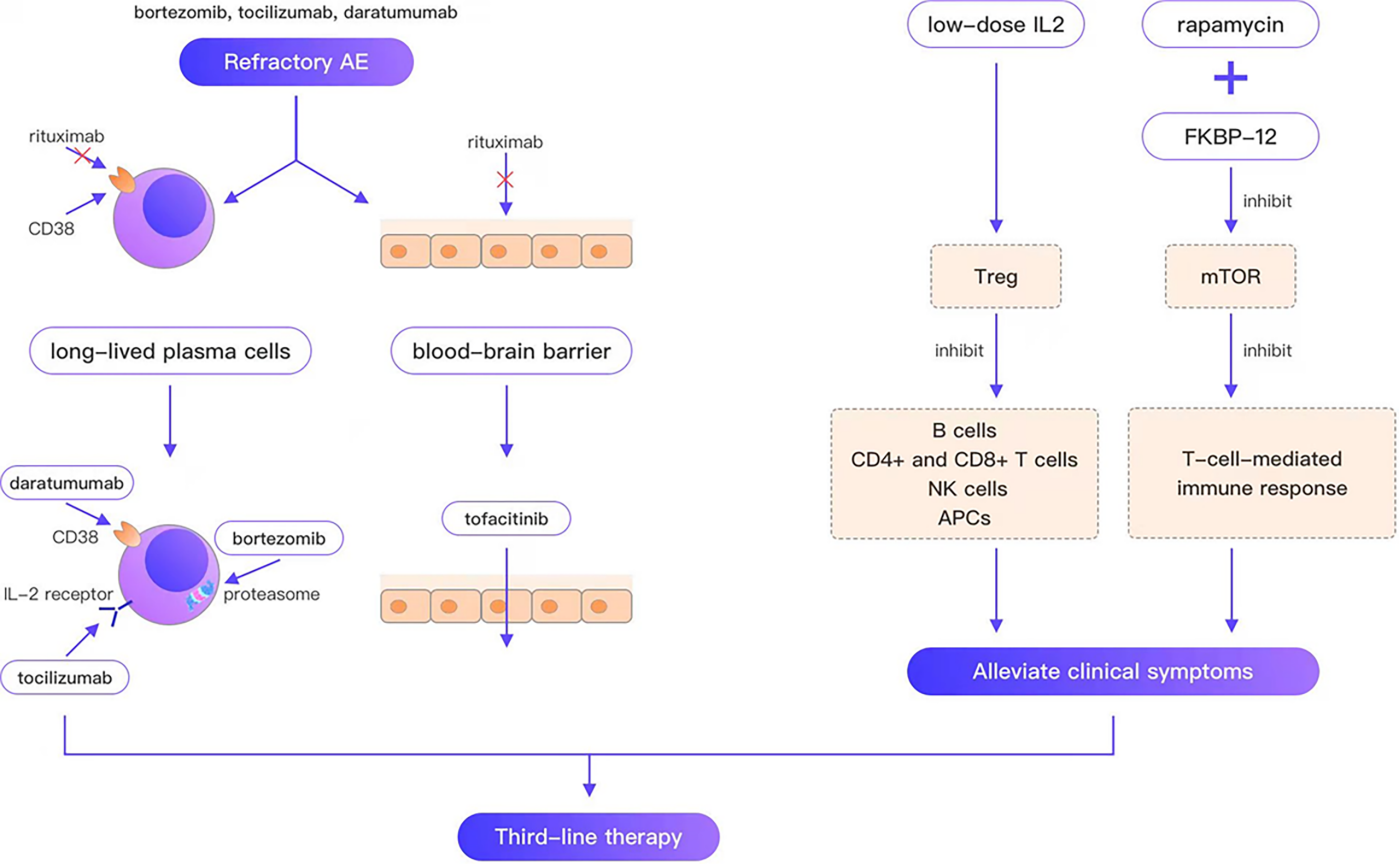
Bortezomib, tocilizumab, and daratumumab overcome the weakness that rituximab cannot target plasma cells; tofacitinib overcomes the weakness that rituximab cannot cross the blood-brain barrier; low-dose IL2, rapamycin can alleviate the clinical symptom.

Refractory AE is defined as cases that are ineffective with conventional first-line and second-line therapy. Rituximab is the representative drug of second-line therapy; however, the failure of rituximab provokes the consideration of third-line therapy for such refractory cases. Since long-lived plasma cells do not express CD20, are unaffected by rituximab, and continue to produce autoantibodies after depleting pre-B cell and mature B cell pools, they are thought to be the main reason for the failure of AE therapies. In a case report of a young woman who died of refractory anti-NMDAR encephalitis, CD19 + lymphocytes comprised only 0.1% of the blood cells in patients 33 and 56 days following rituximab treatment. Autoantibodies were still present in serum after 66 days of rituximab treatment. The autopsy report revealed no B cells, plasma cells, or plasmablasts in any brain region, whereas examination of lymph node tissue revealed many plasma cells aligned along the sinusoidal line. These plasma cells are considered the best explanation for the continued production of antibodies (39). Moreover, peripherally activated B cells can cross the blood–brain barrier (BBB) (40), undergo clonal proliferation, and differentiate between daughter cells into antibody-secreting plasma cells. In contrast, rituximab cannot cross the BBB, which is considered another reason for the failure of rituximab. Consequently, third-line therapies for refractory AE can be divided into three categories (**Figure 1**): (i) treatments targeting long-lived plasma cells, such as daratumumab (targeting CD38, a type II glycoprotein highly expressed on long-lived plasma cells), bortezomib and tocilizumab (negatively affecting plasma cell survival; (ii) immunotherapeutic drugs that can pass through the BBB, such as tofacitinib; (iii) drugs that alleviate clinical symptoms by regulating immune balance and ameliorating neuronal damage, such as low-dose IL-2, and rapamycin.

## Bortezomib

Bortezomib acts as a reversible inhibitor of the 26S proteasome, the endpoint of the ubiquitin-proteasome system and plays a key regulatory role in cellular homeostasis through the degradation of intracellular proteins. It was first used for the treatment of myeloma (41, 42). Once the Bortezomib mechanism of action is established, it is used to treat many other diseases, such as post-transplant desensitization, refractory hematologic diseases, inflammatory injury in intracerebral hemorrhage, and severe viral encephalitis (43–48). The proteasome inhibitors can induce cell-cycle arrest and apoptosis in plasma cells by accumulating the misfolded proteins. Therefore, they can be used to prevent the production of autoantibodies secreted by plasma cells and improve the therapy of autoimmune diseases, such as autoimmune hemolytic anemia (AIHA), immune thrombocytopenia, antineutrophil cytoplasmic antibodies (ANCA) associated vasculitis, immunologic thrombocytopenic purpura (ITP), systemic lupus, neuromyelitis Optica, and particularly refractory AE.

The therapeutic effect of bortezomib to treat refractory AE has been reported in several cases. A previous study reported that a 22-year-old woman with refractory anti-NMDAR encephalitis failed to respond to PLEX and a high dose of rituximab but recovered swiftly after bortezomib treatment (25). Another study described the case of an 8-year-old child with refractory anti-NMDAR encephalitis, despite high-dose of intravenous steroids, intravenous immunoglobulins, and rituximab, who was treated successfully with bortezomib administration (26). After the fourth bortezomib dose, the patient showed successful control of status epilepticus and progressive improvement of consciousness. Eight months later, the child was able to go to school as usual and return to his normal activities. Five severe refractory anti-NMDA encephalitis cases were reported, where patients were in a vegetative state (27). Three patients moved from the vegetative state into minimally conscious states. One patient’s stereotypic movements and rigid behaviors decreased despite no obvious signs of progress into a minimally conscious state. Another patient failed to recover from a vegetative state after two months of bortezomib treatment. The clinical symptoms of a 26-year-old woman showed signs of improvement after receiving bortezomib in combination with tocilizumab and cyclophosphamide to treat refractory anti-NMDA encephalitis (28). In addition, other cases of refractory AE were successfully treated with bortezomib (29).

Bortezomib is well tolerated with minimum adverse reactions. Acute lung injury (49) and bortezomib-associated neuropathy (50) are reported in only a few cases. However, a preclinical study has shown that bortezomib can affect testicular function for a long time (51); that’s why we should pay attention to the reproductive health of male children.

## Tocilizumab

IL-6 is a pleiotropic cytokine that can be synthesized by various cells, including monocytes, macrophages, lymphocytes, keratinocytes, fibroblasts, endothelial cells, and certain kinds of tumor cells (52). IL-6 can induce the differentiation and proliferation of B cells, keep plasma cells alive, induce helper T cell differentiation, and produce other cytokines, such as IL-17 (a cytokine closely related to autoimmune diseases). Furthermore, it can also stimulate cytotoxic T cells, and promote excitotoxicity-induced neuronal damage (53). Tocilizumab, developed in 1993 by a Japanese group led by Tadamitsu Kishimoto of Osaka University, is an anti-interleukin (IL)-6 receptor monoclonal antibody that blocks IL-6-mediated signal transduction (54). Tocilizumab is a humanized anti-IL-6R antibody engineered by grafting the complementarily determining regions of a mouse antihuman IL-6R antibody into a human IgG1k to create a human antibody with a human IL-6R binding site. Tocilizumab binds and neutralizes IL-6R, resulting in the inhibition of various IL-6-mediated biological activities.

Tocilizumab has shown to be effective in treating autoimmune disorders in both adults and children, such as rheumatoid arthritis, neuromyelitis Optica, and multiple sclerosis (55, 56). Moreover, a few cases of treating AE have also been reported, such as a 64-year-old patient with anti-CASPR2 AE switching to tocilizumab after the failure of high-dose steroid and IVIg therapy (30). After one month of treatment, her abnormal behaviors were reduced, and seizures subsided. She exhibited significantly better results in several cognitive assessments, and the titer of anti-CASPR2 antibodies was reduced from 1:1000 to 1:100. After 4 months of treatment, the modified Rankin Scale score of the patient was 0 and she returned to work. A previous study reported three children with refractory AE who showed significant improvement in clinical symptoms with no significant adverse reactions after being treated with tocilizumab (31). In a case report of an 8-year-old girl with anti- glutamic acid decarboxylase (GAD) encephalitis, tocilizumab significantly improved clinical symptoms, while immune globulin, high dose corticosteroids, and plasmapheresis had limited effects (32). In a retrospective study of 91 patients with AE who failed to respond to first-line immunotherapy and rituximab, Lee et al. (33) divided the patients into the tocilizumab, additional rituximab, and the observation groups based on their response to immunotherapy. Their results showed that the MRS score of the tocilizumab group was higher than the other two groups after 2 months of treatment and the last follow-up. After 1 month of treatment, sustained clinical improvement was observed in 89.5% of the patients. They (34) conducted another cohort study and confirmed that the regimen with tocilizumab was more effective than conventional regimens without tocilizumab.

The serious adverse reactions caused by tocilizumab are mostly found in other autoimmune disease cases. This may be related to the fewer cases of AE treated with tocilizumab. For example, in treating a patient with rheumatoid arthritis and another patient with juvenile idiopathic arthritis, meningoencephalitis was induced (57); in treating a patient with refractory giant cell arteritis, tocilizumab was considered to be a possible cause of Pseudomonas meningitis (58). Patients with rheumatoid arthritis who were given tocilizumab also had cognitive impairment and leukoencephalopathy (59). Even so, the treatment itself has the risk of inducing autoimmune encephalitis (60).

Despite the above risks, tocilizumab should be carefully considered in patients who do not respond to first-line and second-line therapy.

## Daratumumab

Daratumumab is a human monoclonal IgGkappa antibody targeting CD38, a 45 kDa type II transmembrane glycoprotein expressed on plasma cells. Daratumumab inhibits the progression of CD38-expressing multiple myeloma tumor cells, inducing apoptosis directly through Fc-mediated crosslinking and triggering immune-mediated tumor cell lysis through complement-dependent cytotoxicity, antibody-dependent cell-mediated cytotoxicity, and antibody-dependent cellular phagocytosis. Therefore, daratumumab could effectively inhibit the growth of CD38-expressing tumor cells and has been approved for treating multiple myeloma since 2015 (61). Since then, it has been applied to treat other tumor diseases such as leukemia, AL amyloidosis, blastic plasmacytoid dendritic cell neoplasm, etc. (62–64). Because CD38 is also utilized on nonmalignant plasma cells, T cells, and NK cells, thus daratumumab is also used to treat other diseases, such as post-transplant treatment and AE, triggered by plasma cells or T cell- and NK cell-mediated immune response (65–68).

The traditional second-line treatment drug rituximab targets CD20, expressed on pre-B cells, immature B cells, mature B cells, and activated B cells but not on plasma cells. Rituximab indirectly inhibits plasma cells’ production by killing B cells and blocking the differentiation of B cells into plasma cells. A substantial fraction of plasma cells, such as long-lived plasma cells, can survive in the absence of B-cell precursors and can continue to produce antibodies that may lead to treatment failure or disease recurrences. Compared with rituximab, the target protein of daratumumab, namely CD38, is expressed on plasma cells, and thus daratumumab can deplete antibody-producing plasma cells to achieve the desired therapeutic effects. In a case report of a 60-year-old patient with anti-CASPR2 encephalitis, the patient started 13 cycles of daratumumab treatment after not responding to methylprednisolone, immunoglobulins, plasma exchange, immunoadsorption, rituximab, and bortezomib for more than five months (35). After 8 treatment cycles, the patient recovered from disorientation, behavioral problems, breathing disorders and went into partial remission. After 12 treatment cycles, the antibody titers in the serum and cerebrospinal fluid of the patients significantly decreased. However, during the 13^th^ treatment cycle, the patient died due to Gram-negative bacterial septic shock. Another study (36) reported a case of a 20-year-old woman with refractory anti-NMDA encephalitis. She received daratumumab after the failure of first-line and second-line therapy and showed clinical improvement. Although only two single cases were reported, it still suggested that daratumumab has potential therapeutic value for AE, but its value should be carefully examined due to its serious adverse events (69).

## Tofacitinib

Tofacitinib is a specific inhibitor of Janus kinase (JAK) 1 and 3. They play an important role in regulating cytokine receptors, pro-inflammatory cytokines, and T-helper cells (70). Tofacitinib was primarily used to treat refractory rheumatoid arthritis (RA) and was approved for medical use by the Food and Drug Administration (FDA) in 2012 (71, 72). Recently, tofacitinib has been employed to treat many other refractory immune-mediated diseases, including psoriasis and ulcerative colitis (73, 74).

In the CNS, the JAK/STAT signaling pathway is mainly associated with gene regulation during development, inflammation, or hormone release. Therefore, tofacitinib is considered a therapeutic candidate for immune-related CNS disorders. However, Experimental autoimmune encephalomyelitis (EAE) models and animal models of multiple sclerosis showed contradictory results (75).

In EAE models, tofacitinib activates tolerogenic dendritic cells that lead to the amelioration of EAE, suggesting a potential therapeutic role in MS (76). However, a low dose of tofacitinib accelerated EAE by activating T17 cells and produced excess interleukin-17 (77). This mechanism can explain the potential for iatrogenic demyelination using this drug (78). However, its ability to pass through the BBB and modulate the immune response to a wide range of cytokine receptors makes it a potential therapeutic candidate to be applied for treating refractory AE (79). In a retrospective study, tofacitinib was administered orally in eight patients with refractory AE. Complete response was observed in two patients, partial response in three patients, and no response in another three patients. It is noteworthy that one of the patients with chronic meningoencephalitis, achieved complete remission after tofacitinib therapy, and magnetic resonance imaging also showed improvement in his brain function. In addition, none of these eight patients treated with tofacitinib experienced serious adverse events, one patient had mild nausea, and one had transient neutropenia (80).

## Low-Dose Interleukin 2(IL-2)

IL-2 was discovered in the supernatants of mice splenocytes cell culture by Morgan et al. in 1976 (81). IL-2 plays an important role in the differentiation, survival, and maintenance of regulatory T cells (also called Tregs). It can activate Tregs without stimulating effector T cells, restoring the balance between regulatory and effector T cells (82).

As the name suggests, regulatory T cells are T cells that regulate or suppress other cells in the immune system. Tregs control the immune response to self and foreign particles (antigens) and prevent autoimmune diseases. Tregs suppress activation, proliferation, and cytokine production of CD4+ T cells and CD8+ T cells, natural killer cells, B cells, and antigen-presenting cells (83).

Initially, IL-2 was administered intravenously in high doses to treat cancer. However, the high dose results in serious adverse events, such as vascular leak syndrome and severe bacteremia. Nevertheless, Tregs have a low activation threshold for IL-2, and the number and function of Tregs are dysregulated in most autoimmune diseases, such as type 1 diabetes, multiple sclerosis (MS), systemic lupus erythematosus (SLE), RA, inflammatory bowel disease, and psoriasis, making low-dose IL-2 a new therapeutic candidate for many autoimmune diseases with low toxic effects (84–86).

A retrospective study demonstrated the efficacy and tolerability of low-dose IL-2 therapy for refractory AE (37). In the study, patients with refractory AE received low-dose of IL-2 at Seoul National University Hospital from October 2015 to March 2016. All four patients with anti-NMDA encephalitis showed good responses to low-dose IL-2 therapy, improving gait speed, memory, language function, and mental status. Among the six patients who are negative for antibodies, two showed a decrease in seizure frequency. Only one serious adverse event was observed, manifested as a severe reduction in neutrophil count, and the patient recovered after subcutaneous injection of colony-stimulating factor. Mild adverse events included two cases of injection site reactions, one case of influenza-like symptoms, one case of nausea, one case of neutropenia, and three cases of subclinical hyperthyroidism.

## Rapamycin

Rapamycin was initially developed as a macrolide antifungal drug, which was banned due to its potent immunosuppressive effect. This situation did not change until mammalian target of rapamycin (mTOR) was discovered. As a natural inhibitor of mTOR, rapamycin’s therapeutic effectiveness was again recognized. Rapamycin inhibits the mammalian target of rapamycin-regulated kinase by forming immunosuppressive complexes with a family of an intracellular protein termed FK506-binding protein 12 (FKBP-12) (87). The ​Rapamycin-FKBP12 complexes limit the cytokine-mediated proliferation of T cells following antigen stimulation, thus inhibiting T-cell-mediated immune responses. In 1997, rapamycin was approved by the US FDA as an immunosuppressive agent, and effectively prevents acute rejection in kidney transplant recipients. Later, it was also used to treat other immune-related diseases.

In recent studies, rapamycin exhibited protective and therapeutic effects in the injured CNS by reducing overzealous inflammatory responses. A previous study reported that the survival rates of mice treated with rapamycin and valacyclovir were higher than those injected with valacyclovir alone, despite similar viral loads in their brain tissues (88). Rapamycin reduced brain lesions, improved motor function, and significantly reduced the secretion of pro-inflammatory cytokines and chemokines in a rat stroke model. These results suggest that rapamycin can alleviate post-stroke inflammation and neuroprotective brain injury (89).

Although rare cases have been reported, rapamycin has been used to treat AE. In a retrospective study, rapamycin was administered in 17 patients with anti-HU-associated encephalitis. Rapamycin improved or stabilized functional disabilities in more than half of patients and managed neurological impairments in two patients (38). In a case of anti-NMDAR encephalitis, rapamycin was used for maintaining treatment because of its long-lasting immunosuppressive effect (26).

## Future Directions and Conclusion

There are many challenges that need to be tackled. The most appropriate timing for initiating third-line therapies, the best choice of doses, and schedules for each drug are not specified. There is still a lack of unified and well-considered guidelines for treating refractory AE, and thus further studies are needed to more precisely and comprehensively assess the advantages and disadvantages of AE therapies and drugs. To date, differentiated antibody therapeutics for the treatment of AE has not been developed. AE is associated with a wide range of antibodies and represents a complex syndrome with diverse clinical manifestations. Different types of AE respond differently to therapeutic agents; for example, patients with LGI1 antibodies respond faster and better to steroids than patients with NMDA antibodies (90). However, uniform treatment (including the same drugs and doses) in differentiated AE patients is still used. Third-line therapy for refractory encephalitis is even more “indistinguishable” for different types of encephalitis. The “effectiveness “exhibited by third-line therapy is controversial due to the paucity of high-level evidence, such as double-blind randomized controlled trials. On the one hand, the patients who used third-line therapy were mostly mixed with multiple immunotherapy drugs, and it is difficult to completely exclude the effect of other immunotherapy on the outcome; On the other hand, part of the symptoms of AE may be treatment-independent, they have independent natural pathological processes, and the effectiveness of third-line therapy may only be coincidental with the natural pathological process (91). Moreover, if the effectiveness of third-line therapy is difficult to confirm, weighing the benefits and risks of applying this drug becomes challenging. Therefore, comprehensive evidence is needed to clarify the effectiveness of third-line therapy. In addition, the clinical presentation of AE patients at different ages varies significantly: the younger the child, the more difficult it is to recognize specific AE syndromes, and its diagnosis will rely on antibodies and other ancillary tests. Whereas elderly patients are often comorbid with multiple diseases related to their age, causing interference with their clinical manifestations and the diagnosis (90). Developing treatment strategies for different ages that are more suitable for their disease characteristics is critical. The study of genetic susceptibility to AE has been unfolded, and the role of genes in refractory AE deserves more attention (92). If specific genotypes exist, through specific pathways, render patients with AE refractory to first - and second-line therapies, then targeted therapies for specific genotypes become possible. Given the low incidence of encephalitis and difficulty in its early diagnosis, more effort is required to established animal models (93). Unfortunately, only a few therapeutic options are available for AE. Therefore, further clinical studies need to be conducted to identify other possible therapeutic targets and explore more therapeutic strategies for AE.

Efforts are, in fact, underway. Basiliximab, a chimeric mouse-human monoclonal antibody to the α chain (CD25) of the IL-2 receptor of T-lymphocytes, has been used to treat patients with anti-GAD65 AE and achieved promising clinical outcomes, including seizure free (94). Although anti-GAD65 encephalitis as a subclass of autoantibodies against intracellular antigens, differs in terms of pathogenesis and therapeutic focus from the AE discussed in this review, it remains possible that the therapeutic effects of basiliximab, which selectively inhibits activated cytotoxic T cells, may benefit patients with AE who have autoantibodies against cell membranes and ion channels, and maybe a potential therapeutic option.

CD19, as one of the hallmark antigens of B cells similar to CD20, has long been linked to AE and is more significantly expressed in antibody-negative AE (95). Expression of CD19 begins at the pre-B cell stage and is stable throughout B cell development. CD19 is also expressed on all plasma cells and most plasma cells of the bone marrow and secondary lymphoid organs. Unlike CD20, which is expressed on a subset of CD4 + T cells, CD19 is expressed only on B cells. Thus, compared to CD20, CD19 has a broader and more specific pattern of B-cell expression. In animal experiment, Anti-CD19 antibodies showed superior depleting activity than traditional CD20 antibodies against a broad subset of B cells, including autoantibody secreting plasma cells (96). However, whether anti-CD19 antibody can be one of the options for treating AE, needed further exploration.

Furthermore, studies have been carried out to explore the role of IL-1 and IL-17 in the pathogenesis of AE (97–99), to treat febrile seizures in AE patients by IL-1 antibodies (100), or to identify the potential role of tramadol, a non-competitive NMDAR antagonist, in reducing movement disorders (101). These drugs alone may not cure encephalitis, but they may be used as complementary treatment options.

To sum up, the timing of upgrade to third-line therapy is very challenging and clinicians need to balance the risk of severe disease with the risk of treatment side effects (102); third-line therapy still provides a therapeutic option for patients with refractory AE and has shown beneficial results in some of the case reports or cohort studies listed in the current study. With ever-growing attention on refractory AE, we can expect that future studies could potentially elucidate these problems and find more effective therapies for AE.

## Author Contributions

XL: Revise and guide the work. JY: draft the article. All authors contributed to the article and approved the submitted version.

## Conflict of Interest

The authors declare that the research was conducted in the absence of any commercial or financial relationships that could be construed as a potential conflict of interest.

## Publisher’s Note

All claims expressed in this article are solely those of the authors and do not necessarily represent those of their affiliated organizations, or those of the publisher, the editors and the reviewers. Any product that may be evaluated in this article, or claim that may be made by its manufacturer, is not guaranteed or endorsed by the publisher.
